# Emerging role of RNA modification N6-methyladenosine in immune evasion

**DOI:** 10.1038/s41419-021-03585-z

**Published:** 2021-03-19

**Authors:** Xin Lou, Juan-Juan Wang, Ya-Qing Wei, Jin-Jin Sun

**Affiliations:** 1 Department of Hepatopancreatobiliary Surgery, The Second Hospital of Tianjin Medical University, Tianjin, China; 2 Cancer Center, Union Hospital, Tongji Medical College, Huazhong University of Science and Technology, Wuhan, China

**Keywords:** Immunosurveillance, RNA modification

## Abstract

The innate and adaptive immune cells have complex signaling pathways for sensing and initiating immune responses against disease. These pathways are interrupted at different levels to occur immune evasion, including by N6-methyladenosine (m6A) modification. In this review, we discuss studies revealing the immune evasion mechanism by m6A modification, which underlies the retouching of these signaling networks and the rapid tolerance of innate and adaptive immune molecules during disease. We also focus on the functions of m6A in main chemokines regulation, and their roles in promotive and suppressive immune cell recruitment. We then discuss some of the current challenges in the field and describe future directions for the immunological mechanisms of m6A modification.

## Facts

- M6A modification acts by regulating RNA transcript, splicing, processing, translation, decay, and participate in the tumorigenesis and metastasis of multiple malignancies.
- M6A doesn’t result in disease, only the breakout of balance among them lead to higher or lower transcripts and translation.
- Most of regulators of m6A modification could lead to immune evasion, including adaptive and innate immune, though few regulators still need more exploration.
- M6A modification could also regulate the chemokine expression by affecting the immune microenvironment, especially immune evasion.

## Open questions

- Whether some critical mediated-gene, in turn, affect the expression of m6A regulators, when immune signals target the specific m6A regulators to mediate gene-specific regulation.
- Whether changing expression or activity of transcription factor and lncRNA, as well as miRNA could affect the m6A process in immune response though these molecules target a different subset of genes.
- Whether m6A is a single modification that presumably affects all modified transcripts in a similar way.
- How and why are some m6A regulators subjected to regulation processes, and how and why do m6A regulators mediate specific gene expression regulation.

## Introduction

While the term epigenetics was first used and defined to explain the phenomenon of phenotype change with non-DNA variations coined by Conrad Waddington in the 1940s, it has been extended to describe both transient and stable “structural adaptation of chromatin regions so as to register, signal or perpetuate altered activity states”^1^. Epigenetic regulation mainly includes RNA modification, DNA modification, histone post-translational modifications, and chromatin remodeling, which play critical roles in biological and pathological processes by interpreting environmental signals and changing related gene expression. This epigenetic regulation is largely involved in programming gene expression in disease, which could balance immune homeostasis for innate or adaptive immune responses to disease, such as the host against the pathogen and cancer process. On the other side, it induces immune evasion by these diseases, especially RNA modification.

RNA could bear distinct modifications at various residues, of which N6-methyladenosine (m6A) is a key regulator of mRNA turnover and translation. The presence of m6A in cellular mRNAs is first proposed by pioneering studies in the 1970s^2^. Shortly thereafter, the function of m6A modification is shown in studies identifying the presence of m6A to cellular mRNA instability^3^. The sequent founding confirms that the enzyme METTL3 synthesizes nearly all m6A in the mRNA transcriptome in the late 1990s^4^. The m6A is found to be a regulated RNA modification that is required for specific developmental processes and spurred the development of mapping technologies to identify m6A-containing transcripts in order to understand how m6A influences cell differentiation and other essential processes. However, though mapping studies have indicated that some m6A sites might be regulated in a tissue-specific or disease-specific manner, showing regulation of m6A stoichiometry which is the fraction of transcripts that contain the m6A mark at a specific nucleotide position, the exact stoichiometry of specific m6A sites and whether and how this stoichiometry changes in different conditions are not known. Since the original publications of m6A-mapping methods in 2012, additional mRNA modifications have been discovered and mapped including m5C, m1A, ac4C, hm5C, 8-oxo-G, and m7G, but m6A remains the most abundant modification. The m6A is emerging as a widespread regulatory mechanism that controls gene expression in diverse biological and pathological processes.

To date, the role of m6A in regulating mRNA fate and function in various cell types has been widely studied. Lots of m6A mapping studies have been described in gene expression databases, exploring m6A maps in diverse organisms and in response to different signals, drug treatments, and disease states. In this review, we summarize the m6A modification regulators of innate and adaptive immunity, and clarify their roles in immune-related signal transduction. We also focus on the functions of m6A in main chemokines regulation, and their role in promotive and suppressive immune cell recruitment.

### mRNA destined for m6A methylation during cellular fate

**Fig. 1:**
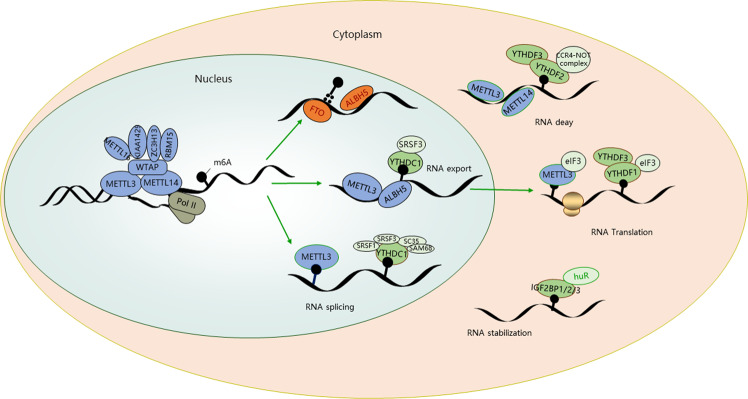
The m6A mRNA life cycle. The m6A modification by writer, eraser, and reader. The figure depicts various writer (blue), eraser (red), and reader (green) proteins involved in the regulation by m6A modification.

The ‘cellular fate’ of an mRNA destined for m6A methylation starts during transcription. The m6A targets the mRNA in the transcription phase. The writing and erasing of m6A mainly pay its function in the nuclear phase, and specific nuclear readers also could affect mRNA splicing, mRNA export, or other nuclear process (Fig. 1). The primary modified RNA was delivered to the cytoplasm, then bind to cytosolic readers, which could mediate the stability, translation, and localization of mRNAs.

### mRNA instability

The best-established function of mRNA instability for m6A was identified by radioisotope metabolic labeling in the 1970s^3^. Another study suggests that the cytosolic m6A-binding protein YTHDF2 could lead to the destabilizing effect of m6A^5^. The METTL3 depletion in diverse cell types and organisms has shown that m6A levels are globally associated with shorter half-lives for modified mRNAs^5^. YTHDF2 selectively binds m6A sites and mediates decay of these transcripts via recruiting the CCR4-NOT deadenylase complex, initiating deadenylation and degradation of targeted transcripts^6^. YTHDF3 may accelerate mRNA decay through interacting cooperatively with YTHDF2^7^. M6A process occurs in the nucleus, which means signaling pathways don’t regulate the m6A levels and function in cytosolic mRNA^8^.

### mRNA translation

The ability of m6A in translation is more complex than in stability, and three distinct ways are found to regulate translation. The first involves the canonical m6A reader YTHDF1 binding to the eukaryotic translation initiation factor eIF3, which recruits the small ribosome subunit to mRNA to enhance the translation. It means that YTHDF1 recruits eIF3 to a location around the stop codon and in 3′ UTR, where m6A RNA could bind to YTHDF1. Another mechanism of m6A-mediated translation enhancement involves direct binding to 5′ UTR m6A to eIF3. The m6A mediated translation initiation does not require eIF4E, and the presence of m6A bypasses the normal requirement for eIF4E, allowing a subset of the m6A-containing mRNAs to be translated when eIF4E is impaired^9^. The third mechanism of translational enhancement involves direct translation activation by METTL3^10^. Interestingly, METTL3 methylates mRNAs in the nucleus but remains bound to the transcript export to the cytoplasm. METTL3 binds to eIF3 in cytoplasm, which could enable ribosomes at stop codons to reload into the 5′ UTR of transcripts^10,11^.

### mRNA splicing

Some of the strongest evidence from studies has confirmed the role of m6A in regulating splicing in Drosophila melanogaster^12,13^. To establish whether m6A regulates splicing in mammals, a major approach has been designed to determine whether m6A is located near exonic or intronic splice junctions, where it could directly influence splicing. Some research groups have shown that m6A is located near the exonic 5 splice site^14^. Others have found m6A near spice site in both exonic and intronic region^13^. Regardless of whether m6A is found in the proximity of splice junctions, most studies have shown that the number of METTL3-dependent splicing events is small^15^. In embryonic stem cells, only a small fraction of known alternatively spliced exons showed altered splicing compared with METTL3-knockout ES cells. These studies suggest that m6A has limited roles in directly controlling mRNA splicing. However, though m6A may only affect splicing in a small number of genes, these m6A-dependent splicing events might be functionally important^16^. Besides, YTHDC1 also was found to be involved, and it interacts with splicing regulators^17^ including SAM68, SC35, SRSF1, and SRSF3, which means m6A might affect the expression of splicing regulatory proteins, and it indirectly regulates the RNA splicing.

### m6A alters innate immunity evasion

**Fig. 2:**
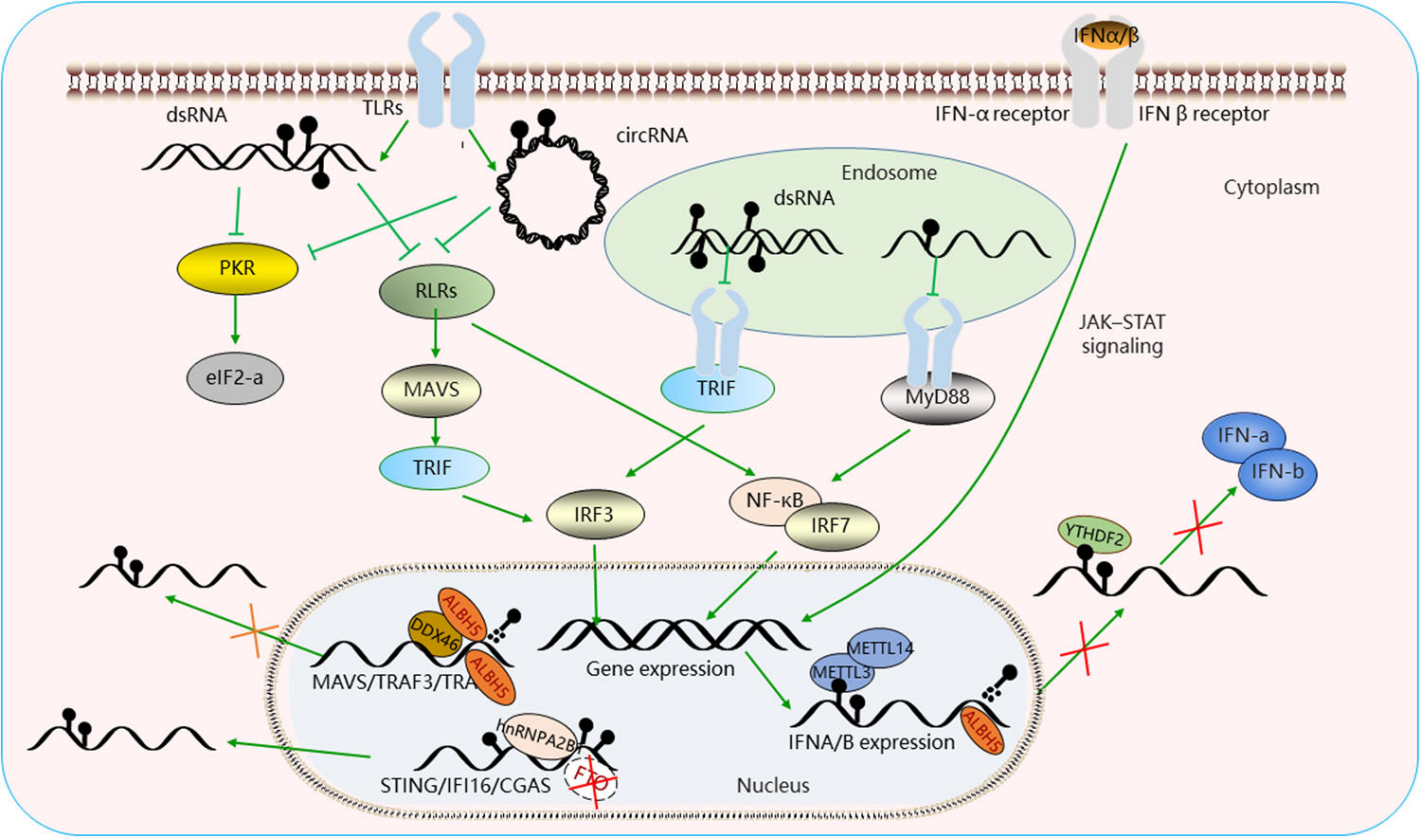
The m6A alters innate immunity evasion. Pathways that are activated by viral RNAs in mammalian cells that express the RLRs, TLRs, PKR, and m6A regulates the process of infection through diverse mechanisms, which increase the risk of immune evasion.

Detection of potential pathogens by innate immunity depends on a subset of encoded recognition receptors, which are both immunogenic and antigenic. There are three major RNA-sensing pathways identified as effective stimulation triggering innate immune, which include cytosolic receptors (RLRs), endosome receptors (TLRs), and protein kinase R (PKR) (Fig. 2). RIG-I could utilize distinct RNA features to discriminate exogenous RNA from endogenous RNAs. RIG-I can recognize viral double-stranded RNAs (dsRNA) that have triphosphate or diphosphate at 5′ ends^18^. The circRNAs are non-coding RNAs generated by back splicing, which produces a highly stable circle structure^19^. Due to the closed circular structure, circRNAs have been used as effective delivery agents, and previous studies have confirmed their immunogenicity^20^. However, the host circRNA avoids immune recognition. M6A modification seems to play a critical role in this process, and mark circRNA as “self”, which could avoid RIG-I recognition and innate immune surveillance by YTHDF2 mediation^20^. Another study has shown that when adenines are replaced with m6A in short interfering RNAs, the immune response against these modified siRNAs also was increasing^21^. There are lots of virus recognized in RIG-I dependent manner, but m6A modification increases the possibility of immune evasion, such as human metapneumovirus. The loss of m6A site or removing m6A from viral RNA could decrease the methylation levels and lead to higher IFN release^22^. Similar to m6A modification, 2′-O-methylation of the exogenous mRNA cap is critical to restrict the actions of IFN-induced proteins resulting in loss of pathogenicity in a mutant absent of 2′-O-methyltransferase activity^23,24^. The interesting study reports that both m6A modification and pseudouridine nucleotides correlate with diminished immune signals response^25^. It also reported the RNA containing m6A modifications binds to RIG-I poorly, while RNA containing pseudouridine binds to RIG-I with high affinity, but both of them failed to trigger the RIG-I conformational changes associated with downstream immune signals^25^. These results suggest that m6A modification interrupts the RIG-I innate immune activation pathway, and that nucleotide modifications with similar chemical structures may be organized into classes, which contribute to avoiding innate immune response. In addition to RIG-I, the MDA5, another RNA receptor of cytosolic receptors, recognizes long RNA absent in the host cell but present in cells infected with RNA viruses^26^. HIV-1 viruses in FTSJ3 knockdown cells show reduction of 2′-O-methylation could induce expression of IFNs in DCs through the MDA5, which identified 2-O-methylation attribute to innate immune evasion in cells infected by HIV-1 viral^27,28^. In addition, TLRs also play a critical role in the immune response. Previous study reports DCs being exposed to m6A modified RNA and sensing the signals by TLR3, TLR7, and TLR8 express significantly less cytokines and activation markers than those treated with unmodified RNA^29^. What’s more, PKR is not a canonical pattern recognition receptor that doesn’t directly induce antiviral or pro-inflammatory cytokine responses, but the protein is important to antiviral activity by inhibiting viral and host mRNAs, leading to suppression of cellular proliferation. A previous study reports that the RNA cellular modifications, such as the m7G cap and m6A group, could inhibit activation of PKR^30^. Cellular human RNAs destabilized by m6A modifications are less folded than once thought, while the non-self RNAs are easily detected because of their unmodified RNAs^30^. A to I editing, another kind of RNA modification, could suppress PKR activity in viral infection^31^, and the proviral role of ADAR1 editing through PKR suppression has been reported in plenty of virus, such as measles virus, human immunodeficiency virus^32^, and human T-cell leukemia virus^33^.

In addition, the production of potent cytokines toward pathogens includes plenty of classification, and we take IFN for example. The sensors trigger signals results in the release of IFNs, including IFNα and IFNβ, which bind to IFN receptor and activate JAK-STAT signals, leading to transcription of ISGs that mediate the antiviral response^34^. The IFN response was regulated by activating and suppressing signals, so the gene expression program that is activated by innate immune recognition has a critical role in this process. RNA modification, especially m6A, may regulate the central mRNA to affect the function. Previous study reports that IFNA and IFNB mRNA encoding cytokines that promote IFN response are modified by m6A, and the loss of METTL3 and YTHDF2 could increase the IFN expression and ISG activity^35^. Another study reported similar results that METTL14 depletion reduced virus reproduction and stimulated IFN expression, but the ALKBH5 depletion had the opposite effect that the ALKBH5 depletion leads to reduction of IFNB expression and increasing of viral propagation^36^. Another study illustrates that TTHDF3 also represses IFN response and ISG expression. The main mechanism is that YTHDF3 enhances the FOXO3 translation to act as the IFN transcription repressor^37^. From what we described, m6A acts as a negative regulator of the antiviral response. However, the other study has the opposite attitude. The Cao group shows m6A promotes antiviral immunity, because the viral infection enhanced DDX46 binding to antiviral proteins MAVS, TRAF3, and TRAF6, which lead to recruitment of ALKBH5. The demethylation leads to retention of these transcripts in the nuclear decreasing their protein levels and inhibiting the production of IFNs. They think m6A plays a critical role in increasing the IFN release through enhancing the export of different components. However, another study reported that hnRNPA2B1, a new receptor to initiate host responses toward herpesvirus infection, could inhibit the recruitment of m6A demethylase FTO to STING, IFI16, CGAS transcript, and amplifies the activation of cytoplasmic TBK1-IRF3 mediated by these factors^38^. Pathogen could improve the IFN release by increasing recruitment of demethylase ALKBH5, while the infection inhibits demethylation by decreasing FTO recruitment. In a short, we still think the m6A modification may have more inhibitory effects on innate immune response, because evaluation in the process must be performed from three potentially interconnected layers of m6A regulation; sensing of foreign RNA, direct regulation of viral transcripts, and regulation of transcripts involved in the cellular response to infection.

### m6A alters adaptive immunity evasion

**Fig. 3:**
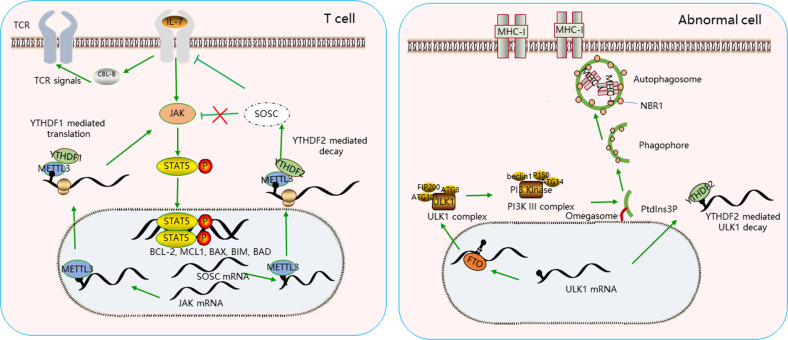
The m6A alters adaptive immunity evasion. The m6A plays a critical role in T cell maturation, differentiation (left); M6A regulates MHC class I appear on the surface of cancer cells by changing the process of autophagy (right).

A recent study shows that A-to-I editing plays a critical role in the maturation and development of T and B cells. Conditional knockout of ADAR1 plays a regulatory role in A-to-I editing via RNA binding domains in T cells in mice. It could result in abnormal thymic maturation of T cells, decreased self-tolerance, and autoimmune symptoms including spontaneous colitis accompanied by diarrhea, bloody stools, and rectal prolaps^39^. The study reports that A-to-I editing indirectly contributed to the adaptive immune system development through the loss of innate immune tolerance to self-RNAs^40^. Similarly, the mechanism by which m6A regulates adaptive immunity is also an emerging field of investigation. The m6A and A-to-I editing, both at adenosines, have an interplay between them. Previous study shows a negative correlation between m6A and A-to-I editing, because the inhabitation of m6A modification increases the association of m6A-depleted transcripts with adenosine deaminase, which could upregulate A-to-I editing on the same m6A-depleted transcripts^41^. In our review, we think the m6A mainly has a positive correlation to adaptive immune evasion (Fig. 3).

### m6A regulation of T cell maturation and differentiation

A previous study has reported that METTL3-deficient CD4 T cells could response to TCR stimulation, and the TCR machinery and downstream signal aren’t associated with m6A modification. We still think m6A mechanism plays an important role in TCR signals in response to antigen recognition on APC. It’s interestingly that METTL14 deficiency was characterized by increased inflammatory cell infiltration, with increase of Th1 and Th17 cytokines, and inhibits naive T cells into induced T_reg_ cells^42^. However, another study suggests that METTL3-deficient T naive cells failed to proliferate under the lymphopenic conditions and are unable to differentiate into pathogenic effector T cells^43^. The conclusion mainly was drawn from the m6A mRNA methylation targeting the IL-7/STAT5/SOCS pathways. IL-7/ STAT5 signal axis is highly important to maintain T cell proliferation and differentiation. The SOCS family (SOCS1, SOCS2, SOCS3, and CISH) could act as mediator binding to IL-7 receptor, then prevent STAT5 activation and downstream signals to regulate T cell maturation, differentiation, so T cells are highly response to IL-7 signals with loss of SOCS genes, while activation of genes suppresses IL-7 dependent T cell. Even so, we still have ourself points. A recent study reported that m6A methyltransferase METTL3 increase the m6A levels of JAK2 and SOCS3 expression, which could enhance YTHDF1-mediated translation of JAK2 and attenuating YTHDF2-dependent mRNA stability of SOCS3^44^. It’s thought that the SOCS3 mRNA stability or half-life was reversed, suggesting that the key genes in the biological process might be regulated by more than m6A regulators. Consistent with these observations, knockdown of METTL3 substantially abolished SOCS2 mRNA m6A modification and augmented SOCS2 mRNA expression, and the study also reports that m6A-mediated SOCS2 mRNA degradation relied on the m6A reader protein YTHDF2-dependent pathway^45^. Moreover, whether the mRNA decay of the SOCS genes is only affected by METTL3 in T cells requires further investigation. Overall, the m6A plays a critical role in T cell maturation, differentiation. In the process, the effect is mediated by regulated by SOCS mRNA stability, and METTL3 may be not alone in the process.

### m6A regulation of antigen-presenting to T cell

Defects in MHC class I protein could lead to immune evasion. Yamamoto et al. demonstrate that suppression of autophagy favors MHC-I re-appearance on the surface of cancer cells because neighbor of BRCA1 gene 1 (NBR1) protein functions as an adaptor to target MHC-I proteins to autophagosomes and autolysosomes in PDAC cells, inducing efficient eradication by CTLs activation^46^, while the upregulated level of m6A modification could induce the activation of autophagy, and the FTO could decrease the m6A modification level of ULK1 to improve autophagy^47^. It means that m6A modification could indirectly depress the MHC class I expression by upregulating the autophagy process in malignant cells. Loss of tumor-associated antigens is another factor to avoid immune recognition. Durable neoantigen-specific immunity is regulated by m6A modification through the m^6^A-binding protein YTHDF1. Loss of YTHDF1 in DC enhanced the cross-presentation of tumor antigens and the cross-priming of CD8 + T cells in vivo^48^. YTHDF1 depletion in DC could decrease the translation of genes related to phagosome and lysosome signals such as enzymes that are members of the cathepsin family^48^. These enzymes could decay proteins in phagosome, which destruct antigens so as to limit antigen cross-presentation in DC^49^. It suggested that higher antigen activation in YTHDF1-deficient DC results in more efficient cross-presentation to CD8 T cells. It’s interesting that knockdown of YTHDF1 could decrease the expression of costimulatory molecular (CD40 and CD80) and inflammatory cytokines, but these defects are not found in DC from YTHDF1-deficient mice^48^. YTHDF1-deficient mice show an elevated antigen-specific CD8 + T cell antitumor response, and a higher level of CD8 cell infiltration was found in patients with loss of YTHDF1 expression^48^. Next, increasing resistance to tumor cell death by immune cells also could induce immune evasion including increased expression of cellular FLICE-like inhibitory protein (cFLIP) or inhibitor of apoptosis proteins (IAPs). NF-κB pathway could positively regulate expression of IAP family, leading to the proliferation and apoptosis inhibition^50^, while up-regulation of RNA demethylase ALKBH5 could improve the activation of the NF-κB pathway in carcinogesis^51^. The RNA demethylase ALKBH5 could increase the IAPs expression by activating the NF-κB signals in tumor cells to resist the immune cells.

### m6A regulation of T cell

CD4 regulatory T cells comprise a critical subset of effector T cells, which are involved in the resolution of inflammation and immunosuppression in tumor microenvironments. The hallmark of Treg cells is the transcription factor Foxp3, and it also has a higher expression of the IL-2 receptor, which also could be regulated by SOCS family and activate the STAT5^52^. METTL3-deficient mice suffers severe autoimmune disease and dies only a few weeks after birth^53^. Different from total CD4 T cell, the depletion of METTL3 in Treg cells lead to increase in the mRNAs of the SOCS gene family, suppress the IL2-STAT5 signaling pathway, and sustains the Treg suppressive function^53^. In addition, METTL14 deficiency in T cells increased inflammatory cell infiltration, also increased Th1 and Th17 cytokines^42^. METTL14 deficiency in mice could inhibit the naive T cells into induced T_reg_ cells^42^. Disruption of mTOR signal in Treg cells leads to Treg suppressive activity in immune tolerance because foreign signals through the TCR and IL-2 provide major inputs for mTOR activation^54^, while another study reports that the m6A modification could upregulated PI3K/Akt/mTOR signal pathway in cancer^55^. It indirectly indicates that m6A machinery enables efficient mTOR signaling to promote Treg suppressive function. Our results indicate m6A modification specifically targets the same class of genes encoding components of essential signaling pathways in different T cell subtypes, thereby controlling the differentiation of naive T cell and also sustaining the suppressive functions of Tregs.

### m6A regulation of immune checkpoint

Expression of ligands for inhibitory receptors, called immune checkpoint, is also an important factor to immune evasion with the help of m6A modification. The loss of YTHDF1 could enhance the therapeutic efficacy of PDL1 checkpoint blockade in mice, which suggests YTHDF1 as a potential therapeutic target in anticancer immunotherapy. Knockdown of FTO decreases the mRNA and protein of PDL1 in HCT-116 cell, and RNA immunoprecipitation assay revealed the m6A modification of the PDL1 and the binding of FTO to the PDL1 in HCT-116, which explained the FTO regulated PDL1 in cell level and provide new insight the regulation of PDL1 by m6A modification^56^. The crucial role of FTO as a m^6^A demethylase in promoting melanoma tumorigenesis and anti-PD-1 resistance is because the combination of FTO inhibition with anti-PD-1 blockade may reduce the resistance to immunotherapy in melanoma^57^. Besides, inhibition of METTL3 and METTL14 could enhance the response to anti-PD1 treatment in melanoma, and tumor lack of METTL3 and METTL14 increases the cytotoxic tumor-infiltrating CD8 T cells and elevates secretion of IFN-γ, CXCL9, and CXCL10 in tumor^58^. Thus, m6A modification could directly target PD1 or PDL1 to regulate the expression, thereby promoting immune evasion functions.

### m6A regulation of chemokine in immune evasion

**Fig. 4:**
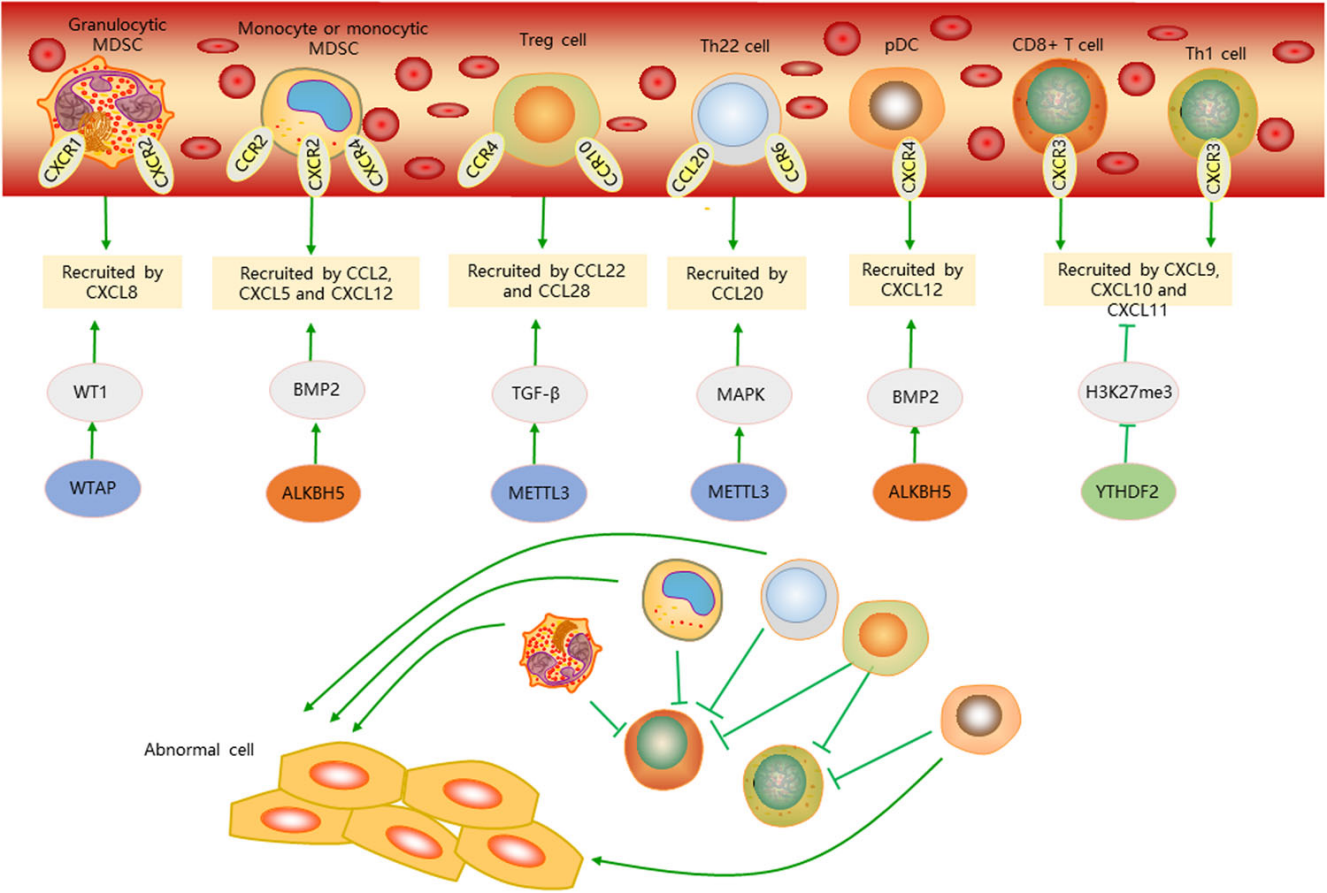
The m6A regulation of chemokine in immune evasion. The m6A could affect the chemokine functions including pro-tumor effects of chemokines involved in Immune cell such as granulocytic and monocytic myeloid derived suppressor cells (MDSCs), Treg cells, Th22 cells, and plasmacytoid dendritic cells (pDCs), and suppress-tumor immunity by chemokines involved in immune cell including CD8 T cell and Th1 cell.

Chemokines are small proteins that are best known for their roles in mediating immune cell trafficking and lymphoid tissue development. The chemokines and chemokine receptors interact to control the migration pattern and location of immune cells. Recent year, chemokine expression regulated by epigenetics is emerging, especially m6A modification (Fig. 4). Aerobic glycolysis is a specific feature of cancer cell metabolism. In aerobic glycolysis, cancer cells produce lactic acid, which activates NF-κB and induces CXCL8 expression in vascular endothelial cells, resulting in angiogenesis in breast and colon cancer^59^. MTHFD2 plays a critical role in controlling global m^6^A methylation levels, including the m^6^A methylation of HIF-2α mRNA. It could result in enhanced translation of HIF-2α and promotes the aerobic glycolysis, which links one-carbon metabolism to HIF-2α-dependent metabolic reprogramming^60^, while hypoxia with hyperactivated HIF-1α and HIF-2α triggers CXCL12 expression in tumor cells^61^, fibroblasts^62^ and haematopoietic stem cells^63^. It indirectly suggested that m6A modification could increase the CXCL12 and CXCL8 expression by activated HIF-2α. In hypoxia of renal cancer, the mechanism of CXCR4 upregulation involves mutation of von Hippel Lindau protein (VHL), while m^6^A RNA demethylase FTO as a synthetic lethal partner of VHL because deletions of FTO are mutually exclusive with VHL loss in pan-cancer datasets^64^. It may imply that FTO could indirectly regulate the CXCR4 expression. In a short, our founding confirmed that the m6A modification could also regulate the chemokine expression by affecting the metabolism environment.

Effector T cells, NK cells and NKT cells could mediate an antitumor immunity, whereas Treg cells and Th22 cells promote tumorigenesis. Th22 cells are found in the microenvironment of several types of human cancer, including colon cancer, pancreatic cancer and hepatocellular carcinoma^65^. The cells express CCR6, migrate towards the CCR6 ligand CCL20 in cancer microenvironment, and have been shown to promote and support tumorigenesis^65^. Th22 cell-derived IL-22 acts on cancer cells to promote the activation of the transcription factor signal transducer and activator of STAT3, increase the expression of the H3K79 methyltransferase DOT1L30, and upregulate the expression of the H3K27 methyltransferase PRC2, particularly the enhancer of EZH2 subunit. The DOT1L complex induces the expression of the core stem cell genes NANOG, SOX2 and POU5F1, resulting in increased cancer stemness and tumorigenic potential^65^. Treg cells express CCR4 and are recruited into the tumor microenvironment in response to CCL22, which is produced mainly by macrophages and tumor cells^66^. In addition to the CCL22–CCR4 signaling pathway, Treg cells express CCR10 and migrate in response to the CCL28 that is found in hypoxic tumor microenvironment^67^. These Treg cell populations are recruited into the bone marrow via the CXCL12–CXCR4 signals and are expanded by DCs by the receptor activator of NFκB–RANK signals, which also increase the immune evasion.^68^

It is indispensable for TGF-β-induced EMT in lung cancer cells, and METTL3/m^6^A-mediated TGF-β signaling have been confirmed^69,70^, while the elevated TGF-β activity could lead to enhanced production of chemokine CCL22 by suppressing expression of microRNA-34a^71^, which recruits Treg cells to facilitate immune escape. The previous study has confirmed that the expression of IL-6, IL-8, and CCL20 were mediated by activation of MAPK signaling^72^, the phosphorylation levels of which were reduced upon methyltransferase METTL3 expression deficiency^73^. It may indicate that METTL3 could increase the infiltration level of Th22 cell by MAPK-CCL20 signal. In addition, Wilms’ tumor protein 1(WT1) could function as a mitotic transcription factor and specify CXCL8/IL-8 as a target gene of WT1 that conveys mitotic survival^74^, while Wilms’ tumor 1-associating protein (WTAP) has been identified as a key subunit of the m6A methyltransferase complex, which indirectly identify the WTAP could regulate the MDSC infiltration to affect the immune environment.

## Conclusions and perspectives

M6A modifications act by regulating RNA transcript, splicing, processing, translation, decay, and participate in the tumorigenesis and metastasis of multiple malignancies. Since m6A modification and the machinery have been implicated in the initiation, progression, maintenance, and drug resistance of various types of disease, the research of m6A regulator in immune response is still in infancy. M6A modification could support rapid phenotypic variation for disease. In the disease, m6A regulators from pathogen contribute to alter transcription patterns of pathogen expression, which could result in immune evasion by the pathogen and persistence in the other disease. However, these meaning conclusions were drawn according to the deletion of one of regulators of m6A modification, and the other mRNA or molecular components methylation levels associated with critical phenotype remain to be explored.

Several major issues should be further investigated to fully appreciate the function of m6A regulation in immunity response to disease. 1) the majority of immunological phenotypes that depend on m6A were studied using cells in which one of the essential components of the m6A regulator complex was deleted. Identifying which of the m6A regulators drives the observed phenotypes, whether other regulator also co-regulatory result remained to be investigated. Since some of the m6A regulators have different function in mRNA transcript, for example, FTO, m6A ‘erasers’, which are demethylases that convert m6A into A, also shape the m6A epitranscriptome, so their effects on phenotypes may have a variation compared with m6A writer, even have opposing trend. In addition, YTHDC1 and YTHDF1 were suggested to enhance expression of m6A-mediated transcripts by stimulating export from nuclear and translation, while the YTHDF2 was reported to increase the stability and decay of m6A-mediated transcripts. Understanding whether there are corresponding opposing effects of these regulators will provide us a better know of complex process. The phenotypic dissection can be extended to molecular analysis to understand the mechanism through how the m6A reader regulated with the cellular. We also suspect whether the disease attribute to the out-of-balance effect among the m6A regulator opposing effects on normal protein regulation, such as YTHDF1 and YTHDF2. In other words, the m6A didn’t result in disease, only the breakout of balance among them lead to higher or lower transcripts and translation.

In the context of immune regulation, how immune signals target the specific m6A regulators to conduct gene-specific regulation. Some of m6A-mediated transcripts are immune-related genes which suggested to drive phenotypic changes in the immune system. We found the m6A function in immunosuppression environment seems to more activated compared to immune promote environment. The m6A modification could activate more Treg cells, and the m6A modification chemokine could recruit more Treg cells. Of course, the phenomenon also found in TAM, and MDSC. However, the m6A modification has also been reported to promote the immune response, but we think the m6A regulator plays a critical role in the divergence. At present, of all m6A regulators, only METTL3 was found to could stimulate the T differentiation and proliferation, but we also have the different opinions that we think the other regulator YTHDF2 could decay the SOCS genes, which seem to hardly prevent the JAK/STAT5 signals. In addition, other m6A regulators all have been reported to stimulate immune evasion. Furthermore, the m6A modification could repress the MHC I expression on the surface of cancer cells, which means the tumor could avoid the innate and adaptive immune response. The m6A regulator FTO also could upregulate the immune checkpoints, which also promote the immune evasion. The m6A seems to increase the activity of immunosuppressive cells and inhibit the activity of immune-promoting cells. The same results also were found in chemokine. We found the Treg-type chemokine was activated by m6A modification, and the Th1-type chemokine was inhibited. Of course, we think the m6A modification in immune evasion needs further exploration, and the reading, writing, and erasing mRNA methylation on immune signals needs to be explored, respectively.

In the innate immune response, we emphasis the viral or pathogen in the body, and three inter-connected layers of m6A modification, including sensing of foreign RNA, direct recognition of viral transcripts, and regulation of transcripts. It is clear that m6A modification regulates foreign RNA and key transcripts in the type I IFN response, then we discuss the role of m6A in sensing foreign RNA and how this process affects the downstream and observed phenotypes. The sensors including RLRs, TLRs, and PKR are all mediated by m6A, and become more sluggish in response to external environment changes, which increase the risk of immune evasion. Maybe direct measure of foreign or cellular RNA at specific cells lack of m6A machinery could be informative for underlying mechanisms.

Though plenty of advances have been made in the field of m6A modification, our understanding of how m6A modification affects the immune response, especially immune evasion remains a blank. Our review mainly discusses the m6A process, it is becoming apparent that the collection of modifications on mRNAs might be wider. At last, open questions remain: how and why are some m6A regulators subjected to regulation processes described above, and how and why do m6A regulators mediate specific gene expression regulation.
